# NANO-GBM trial of AGuIX nanoparticles with radiotherapy and temozolomide in the treatment of newly diagnosed Glioblastoma: Phase 1b outcomes and MRI-based biodistribution

**DOI:** 10.1016/j.ctro.2024.100833

**Published:** 2024-07-31

**Authors:** J. Biau, X. Durando, F. Boux, I. Molnar, J. Moreau, B. Leyrat, F. Guillemin, A. Lavielle, Y. Cremillieux, K. Seddik, S. Dufort, O. De Beaumont, E. Thivat, G. Le Duc

**Affiliations:** a Radiation Department, Centre Jean Perrin, Clermont-Ferrand F-63011 France; b University of Clermont Auvergne, UFR Médecine, Clermont-Ferrand, France; c INSERM U1240 IMoST, University of Clermont Auvergne, Clermont-Ferrand, FR-63000, France; d Centre d’Investigation Clinique UMR 501, Clermont-Ferrand F-63001, France; e Department of Clinical Research, Délégation Recherche Clinique et Innovation, Centre Jean Perrin, F-63011 Clermont-Ferrand, France; f Oncology Department, Centre Jean Perrin, Clermont-Ferrand F-63011 France; g NH TherAguix SA, Meylan, France; h Institut des Sciences Moléculaires, UMR5255, Université de Bordeaux, France

**Keywords:** Glioblastoma, Radiotherapy, Nanoparticles, AGuIX

## Abstract

**Background:**

In glioblastoma (GBM), tumor progression occurs mainly within the irradiated tumor volume. To address this challenge, a radiosensitization strategy with intravenous gadolinium-based theranostic nanoparticles (AGuIX) is being explored in the NANO-GBM phase1b/2R trial (NCT04881032). Here, we present the results of the phase 1b part, which is the first-in-human use of these nanoparticles with radiotherapy and chemotherapy for the treatment of newly diagnosed GBM.

**Material and Methods:**

Eligible patients were aged 18 to 75 years with newly diagnosed and histologically confirmed GBM, with incomplete resection (biopsy or partial surgery). The phase 1b part was a dose escalation approach (Time-to-event Continuous Reassessment Method) with three dose levels: 50, 75, and 100 mg/kg. Patients were treated with RT (60 Gy), and concomitant and adjuvant TMZ, and 4 injections of AGuIX (D-3/-7, D1, D8, and D15). Dose-limiting-toxicity (DLT) was defined as any grade 3–4 adverse event (CTCAE v5.0), excluding alopecia, nausea, and rapidly controlled vomiting. Pharmacokinetic (PK), and biodistribution based on MRI were evaluated.

**Results:**

Between March 2022 and March 2023, eight patients were enrolled: 1 at 50 mg/kg, 1 at 75 mg/kg, and 6 at 100 mg/kg. All patients received the four AGuIX injections. Only one patient experienced a DLT (at 100 mg/kg): a grade 3 lymphopenia (related to TMZ). The RP2D of AGuIX was determined as 100 mg/kg. AGuIX mean AUC increased with dose. Regions of GBM with moderate (36–123 µM), and high (123–291 µM) or very high (>291 µM) AGuIX concentrations accounted in average for 38.7 and 26.8 %, respectively.

**Conclusion:**

These results confirm the lack of AGuIX-related toxicity and the widespread dispersion of nanoparticles throughout GBM. This supports progression to the randomized phase 2 part, utilizing an RP2D of AGuIX of 100 mg/kg (4 injections).

## Highlights

• Phase 1b trial of the combination of intravenous AGuIX nanoparticles with RT and TMZ in GBM patients.
• First-in-human phase 1b trial to evaluate this combination.
• No DLT related to AGuIX were reported.
• The RP2D dose of AGuIX was 100 mg/kg with chemoradiotherapy.
• Selective accumulation of AGuIX nanoparticles in tumor tissue.

## Background

Glioblastoma (GBM) is the most common and aggressive brain tumor in adults [1]. Although radiotherapy (RT) with concomitant and adjuvant temozolomide (TMZ) +/- tumor-treating electric fields has increased patient survival, long-term prognosis remains poor, with a median survival of 15–21 months [2], [3]. Tumor progression occurs in approximately 90 % of cases within the irradiated tumor volume [4], demonstrating the high radioresistance of these tumors. To address this challenge, a tumor-specific radiosensitization strategy with nanoparticles is being explored in the NANO-GBM phase1b/2 trial (NCT04881032) [5]. Only two types of nanoparticles have been clinically evaluated to date for their radiosensitizing properties. The first is NBTXR3 hafnium oxide (HfO2) nanoparticles, which have demonstrated efficacy in the treatment of patients with locally advanced soft-tissue sarcoma in an international, randomized, phase 2/3 study. Intra-tumoral injection of NBTXR3 nanoparticles prior to preoperative RT significantly increased the complete response rate (16 % vs. 8 %, p = 0.044) compared with RT alone (15). The second type is AGuIX nanoparticles (Activation and Guidance of Irradiation by X-ray), a gadolinium-based theranostic nanoparticle that are administered intravenously. This advantage is particularly important in neuro-oncology where intra-tumoral injections are challenging. AGuIX nanoparticles have shown promise as effective radiosensitizers and contrast agents [6]. Preclinical studies on orthotopic tumor models, including GBM and brain melanoma metastases, have demonstrated improved survival outcomes when AGuIX nanoparticles are combined with RT and TMZ [6], [7], [8]. Accumulation of AGuIX in GBM has been observed, along with its prolonged retention in tumor tissues due to the Enhanced Permeability and Retention (EPR) effect [9]. A first phase 1b trial has already shown a favorable toxicity profile of AGuIX when combined with whole-brain RT for the treatment of multiple brain metastases (NANO-RAD, NCT02820454) [10]. Here, we report the results of the phase 1b part of the NANO-GBM trial, which is the first-in-human use of AGuIX nanoparticles with RT and TMZ in the treatment of newly diagnosed GBM with incomplete resection. The primary objective was to determine the recommended dose of AGuIX in combination with standard-of-care TMZ and RT. Secondary objectives were to characterize the pharmacokinetics of AGuIX and its biodistribution based on Magnetic Resonance Imaging (MRI).

## Materials and methods

### Patients

Eligible patients were aged 18 to 75 years with newly diagnosed and histologically confirmed GBM, having undergone incomplete resection (biopsy or partial surgery), with Karnofsky Performance Status (KPS) ≥ 70, and adequate hematologic and hepatic function. Patients receiving corticosteroids were eligible if they had been on a stable or decreasing dose for at least 14 days before enrollment.

Patients with a history of other malignancy within 5 years prior to enrollment, contraindication to MRI or gadolinium injection, and/or concurrent administration of immunosuppressive therapy were excluded. All patients provided written informed consent before enrollment.

### Study design and treatment plan

The NANO-GBM trial is a multicenter, phase 1b/2, randomized, open-label, non-comparative, therapeutic study [5]. The study protocol was approved by the ethics committee and the national regulator and registered with https://www.clinicaltrials.gov (NCT04881032). In this article, we exclusively focus on the results of the phase 1b part of the NANO-GBM trial. The phase 1b part consists of a dose escalation phase involving three dose levels of AGuIX: 50 (dose 1), 75 (dose 2), and 100 (dose 3) mg/kg. The dose escalation was guided by a Time-to-event Continuous Reassessment Method [11] (for more detail see the published protocol [5]).

**Fig. 1:**
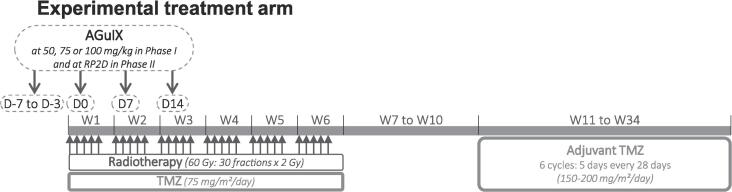
Overview of the treatment period of NANO-GBM phase 1b.

Patients were treated with RT and concomitant and adjuvant TMZ [2], along with the addition of AGuIX nanoparticles during the concomitant period (Fig. 1).

***Radiotherapy.*** For irradiation, all patients were immobilized using custom thermoplastic masks. A planning MRI was performed within 5 to 15 days before the initiation of RT. Target volume and organs at risk delineation were conducted using a dedicated CT-scan in the treatment position, which was matched and fused with contrast-enhanced T_1_-weighted and Fluid-Attenuated Inversion Recovery (FLAIR) MRI sequences. The gross tumor volume (GTV) was defined as the area of contrast enhancement in the T_1_-weighted MRI sequence, including the tumor bed for patients with partial resection. The clinical target volume (CTV) was defined as the addition of a geometric tridimensional 1-cm margin around the GTV, which was adjusted to the anatomical borders [12]. The CTV also encompassed hyperintensity in the FLAIR MRI sequence. The planning target volume (PTV) was defined as the addition of a geometric tridimensional 4-mm margin around the CTV. RT was administrated once daily, five days per week over a period of 6 weeks, with a total dose of 60 Gy in 30 fractions of 2 Gy each. All patients underwent treatment with volumetric modulated arctherapy (VMAT).

***Temozolomide*.** Concomitant TMZ at a dose of 75 mg/m^2^ per day was administrated 7 days per week from the first day of RT until the end of RT. Four weeks after RT, patients received a maximum of 6 cycles of adjuvant TMZ according to the standard 5-day schedule every 28 days (at a dose of 150 mg/m^2^/day for the first cycle and then at 200 mg/m^2^/day in the absence of toxicity).

***Experimental radiosensitizer AGuIX.*** The experimental treatment involved the administration of AGuIX nanoparticles, provided by NH TherAguix (Meylan, France), via intravenous injections (injection rate: 2 mL per minute). AGuIX nanoparticles measure approximately 5 nm in diameter and consist of polysiloxane network grafted with about 15 Gadolinium chelates [13]. Each patient received a total of four injections. The first injection was administered 3 to 7 days before the initiation of RT. Subsequent injections were administered during RT, specifically on the first day of weeks 1, 2, and 3. The injections were administrated approximately 4 h (±1 h) before RT and 3 h before TMZ administration. Three dose levels of AGuIX (50, 75, and 100 mg/kg) were evaluated in the phase 1b.

### Assessments

***Safety.*** Toxicity was assessed weekly during the concomitant phase (RT+TMZ+AGuIX) and then monthly during the adjuvant phase (TMZ alone), through physical examination, hematologic and chemistry laboratory values, and vital signs. Adverse events (AE) were graded according to the National Cancer Institute’s Common Toxicity Criteria version 5.0 (NCI-CTCAE v5.0). Dose Limiting Toxicity (DLT) was defined as any grade 3–4 AE, excluding alopecia, nausea, and vomiting, which could be rapidly controlled with appropriate measures. Only toxicities occurring between the first injection of AGuIX until the end of the concomitant phase (RT+TMZ+AGuIX) were considered for DLT assessment.

***Pharmacokinetic assay.*** Gadolinium concentration, representative of AGuIX concentration, was measured in plasma and urine samples using Inductively Coupled Plasma – Mass Spectrometry (ICP-MS) [13]. Whole blood samples were collected on the second, third and fourth injection with a 9-point kinetic analysis over 24 h. A 6-hour urine collection was also conducted. Pharmacokinetic analyses were performed using WinNonLin Professional Version 6.4.0.768 (Pharsight Corporation, Mountain View, California, USA) including the area under the plasma concentration/time curve to the last sampling time point (AUC), maximum measured plasma concentration (Cmax), half-life (T_1/2_), plasma clearance (CL), and volume of distribution (Vss).

**Fig. 2:**
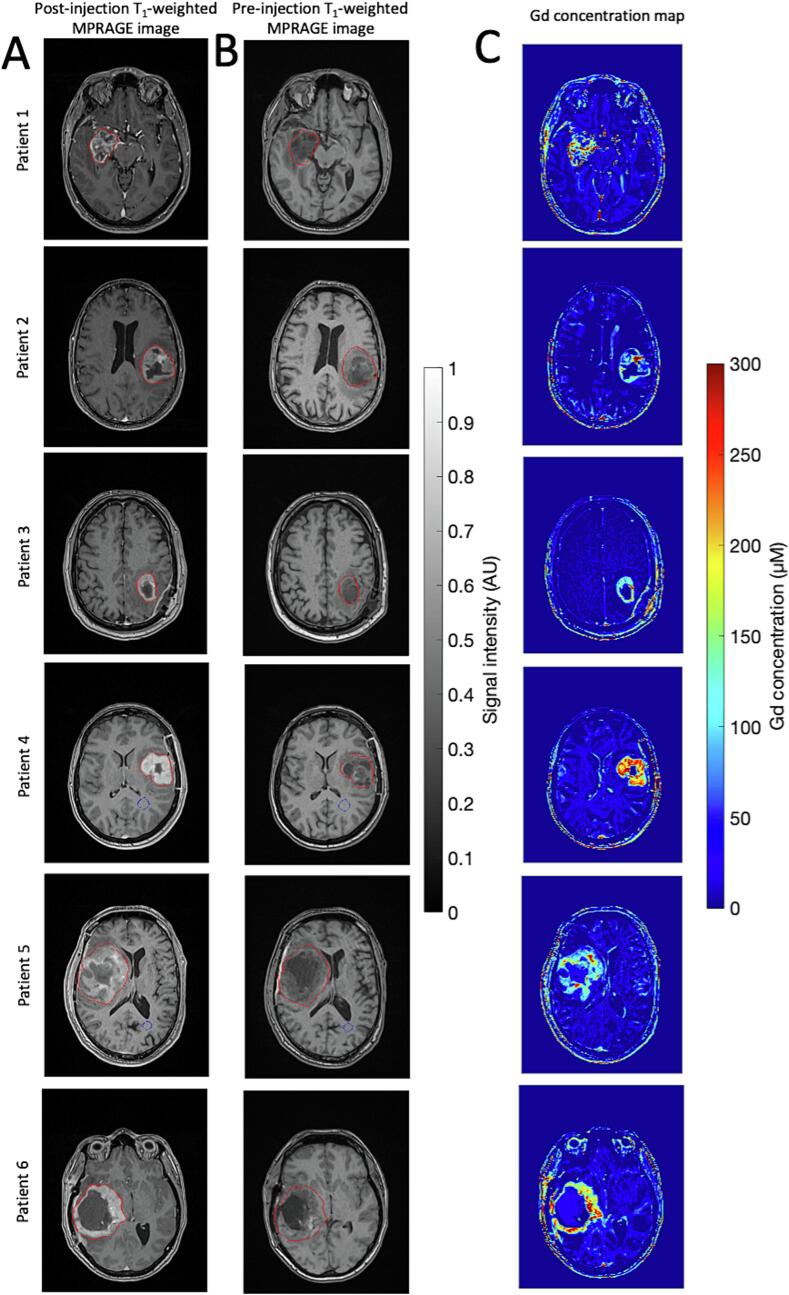
**MRI-based biodistribution of AGuIX of patients treated at RP2D (100 mg/kg, n = 6). A.** T_1_-weighted MPRAGE image obtained approximately 1 h after intravenous administration of AGuIX nanoparticles at a dose of 100 mg/kg body weight. This image is used to identify specific regions of interest: glioblastoma (*red contour*), healthy occipital white matter (*blue contour*), and vitreous body eyeball (not visible in these images). **B.** Co-registered T_1_-weighted MPRAGE image acquired prior to the administration of nanoparticles. **C.** Concentration map of gadolinium (Gd^3+^) from AGuIX nanoparticles. To enhance clarity, the color scale for Gd^3+^ concentration maps is truncated between 0 and 300 µM, with values exceeding this range adjusted to the respective limits. For images and maps, the field of view is 203x250 mm^2^ with a resolution of 0.49 x 0.49 mm^2^. (For interpretation of the references to color in this figure legend, the reader is referred to the web version of this article.)

***Biodistribution based on MRI.*** Two additional MRI were performed within a range of 1 to 2 h after the completion of the first and fourth AGuIX injections (see Supplementary Material Table S1 for patient-specific timing) to assess its biodistribution, using a 1.5 T MAGNETOM Sola scanner equipped with a Head/Neck 20 MR coil for signal reception (Siemens Healthcare, Erlangen, Germany). T_1_-weighted imaging was acquired using a 3D Magnetization Prepared − RApid Gradient Echo (MPRAGE) sequence with a field of view of 203 x 250 x 176 mm^3^ and resolution of 0.49 x 0.49 x 1.00 mm^3^. For image processing and delineation of regions of interest (ROI), we used the Olea SDK software (version 1.5.3; Olea Medical, La Ciotat, France). Four ROIs were manually delineated for each patient: tumor, healthy occipital white matter, healthy occipital gray matter, and vitreous body eyeball region. The computation of T_1_ maps from MPRAGE sequences was carried out using a theoretical approach described in Lavielle et al. [14]. This approach relies on an analytical expression of the signal amplitude and incorporates a tissue reference T_1_ value (in this case, gray matter in the occipital lobe with T_1_ = 1100 ms [15]). The quantification of concentration of Gd^3+^ (representative of the presence of AGuIX nanoparticles) requires a prior co-registration of the pre- and post-AGuIX T_1_-weighted imaging sequences (Fig. 2A and B) and the longitudinal relaxivity of AGuIX. The longitudinal relaxivity values were determined based on time-domain nuclear magnetic resonance measurements of as-injected AGuIX solution at 1.5 T and 37 °C (all details in Supplementary Material Table S1). The concentration values in the maps represent the average concentration across the voxel volume, approximately 0.24 mm^3^.

Additionally, maps illustrating the varying levels of Gd^3+^ concentration were created. We used voxels within the GTV ROI from all patients to construct a global histogram. To characterize this histogram, we employed a 4-Gaussian mixture model that captured the characteristic concentration distributions in biological tissues. Subsequently, the Gaussians in the model were sorted, and an automatic Otsu's method was applied to determine the optimal threshold that effectively separates two consecutive Gaussians while minimizing intra-class intensity variance [16]. These determined thresholds were then used to define the ranges of concentration used for classifying voxels.

### Statistical considerations

The primary end-point of the phase 1b part was the determination of the recommended phase 2 dose (RP2D) by evaluating the maximum tolerated dose (MTD) of AGuIX. The MTD was defined as the highest dose tested in which a DLT was experienced by no more than 33 % of patients. Patients who received at least one injection of AGuIX were evaluable for safety analysis. Due to the exploratory nature of the current study, and the relatively low number of patients, mainly descriptive statistics were used, including medians, ranges, and Standard Deviations (SD).

## Results

Between March 2022 and March 2023, eight patients were enrolled in the phase 1b part of the study: cohort 1 at dose 1 (50 mg/kg): n = 1; cohort 2 at dose 2 (75 mg/kg): n = 1; cohort 3 at dose 3 (100 mg/kg): n = 6. Patient characteristics at baseline are presented in Table 1. Six patients were male, and two were female. Six patients had a KPS score of 100, one had a score of 90, and one had a score of 80. Five patients had undergone a partial resection and three hade undergone biopsy. All enrolled patients received the four AGuIX injections as planned and completed the concomitant phase (RT+TMZ+AGuIX).

**Table 1:** Demographic and clinical characteristics.

Characteristic	All patients (n = 8)	100 mg/kg (n = 6)
**Age** (years): median [range]	62 [48–66]	63 [53–66]
**Gender**		
Male	6	4
Female	2	2
**Karnofski (%)**		
100	6	5
90	1	1
80	1	0
**Tumor location**		
Frontal	3	2
Temporal	2	2
Parietal	3	2
**IDH**		
Mutant	1	0
Wild type	7	6
**Surgical resection**		
Partial	5	4
Biopsy only	3	2

Demographic and clinical characteristics of patients enrolled in the phase 1b of the NANO-GBM trial.

### Safety

All treatment-related AE experienced during the concomitant phase, and the adjuvant phase are described in Table 2. Patients treated at doses 1 and 2 had no DLT. Among the six patients treated at dose 3, one presented a DLT: grade 3 lymphopenia 36 days after the first AGuIX injection, considered unrelated to the experimental treatment but to standard TMZ chemotherapy. There were no grade 4 or 5 AE. One patients experienced a grade 3 AE after the DLT assessment period: one lymphopenia at day 107. Based on observed DLTs, the dose of 100 mg/Kg of AGuIX was determined as the RP2D. During the concomitant phase (RT+TMZ+AGuIX), the most frequent treatment-related AE were: grade 2 increased monocyte count (n = 6, 75 %), hypoglycemia (n = 5, 62.5 %), hyperglycemia (n = 3, 37.5 %), fatigue (n = 3, 37.5 %), and headaches (n = 3, 37.5 %). The only AE that could be directly attributable to AGuIX was one (12.5 %) grade 1 injection site hematoma.

**Table 2:** Treatment-related adverse events.

	Subjects experiencing a treatment-related adverse event, *n (%)*							
	Radiation phase				Adjuvant phase			
Grade*	1	2	3	4_5	1	2	3	4_5
Hematologic								
Anemia	0 (0)	0 (0)	0 (0)	0 (0)	1 (12.5)	0 (0)	0 (0)	0 (0)
White blodd cell count increased	1 (12.5)	0 (0)	0 (0)	0 (0)	0 (0)	0 (0)	0 (0)	0 (0)
White blodd cell count decreased	0 (0)	0 (0)	0 (0)	0 (0)	0 (0)	0 (0)	0 (0)	0 (0)
Lymphocyte count decreased	1 (12.5)	0 (0)	1 (12.5)	0 (0)	0 (0)	1 (12.5)	1 (12.5)	0 (0)
Neutrophil count increased	1 (12.5)	0 (0)	0 (0)	0 (0)	1 (12.5)	0 (0)	0 (0)	0 (0)
Neutrophil count decreased	0 (0)	0 (0)	0 (0)	0 (0)	0 (0)	0 (0)	0 (0)	0 (0)
Monocyte count increased	6 (75)	0 (0)	0 (0)	0 (0)	0 (0)	0 (0)	0 (0)	0 (0)
Platelet count decreased	1 (12.5)	0 (0)	0 (0)	0 (0)	0 (0)	0 (0)	0 (0)	0 (0)
Serum chemistry								
Hyperglycemia	3 (37.5)	0 (0)	0 (0)	0 (0)	2 (25)	1 (12.5)	0 (0)	0 (0)
Hypoglycemia	5 (62.5)	0 (0)	0 (0)	0 (0)	5 (62.5)	0 (0)	0 (0)	0 (0)
Hypoprotidemia	2 (25)	0 (0)	0 (0)	0 (0)	6 (75)	0 (0)	0 (0)	0 (0)
Hypernatremia	2 (25)	0 (0)	0 (0)	0 (0)	2 (25)	0 (0)	0 (0)	0 (0)
Hyponatremia	1 (12.5)	0 (0)	0 (0)	0 (0)	0 (0)	0 (0)	0 (0)	0 (0)
Hypokalemia	2 (25)	0 (0)	0 (0)	0 (0)	0 (0)	0 (0)	0 (0)	0 (0)
ALT increased	2 (25)	0 (0)	0 (0)	0 (0)	0 (0)	0 (0)	0 (0)	0 (0)
AST increased	1 (12.5)	0 (0)	0 (0)	0 (0)	0 (0)	0 (0)	0 (0)	0 (0)
Gamma-glutamyl transferase increased	1 (12.5)	0 (0)	0 (0)	0 (0)	0 (0)	0 (0)	0 (0)	0 (0)
Constitutional								
Fatigue	3 (37.5)	0 (0)	0 (0)	0 (0)	0 (0)	0 (0)	0 (0)	0 (0)
Headaches	3 (37.5)	1 (12.5)	0 (0)	0 (0)	1 (12.5)	0 (0)	0 (0)	0 (0)
Nausea	2 (25)	0 (0)	0 (0)	0 (0)	0 (0)	0 (0)	0 (0)	0 (0)
Vomiting	2 (25)	0 (0)	0 (0)	0 (0)	0 (0)	0 (0)	0 (0)	0 (0)
Constipation	0 (0)	0 (0)	0 (0)	0 (0)	1 (12.5)	0 (0)	0 (0)	0 (0)
Injection site hematoma	1 (12.5)	0 (0)	0 (0)	0 (0)	0 (0)	0 (0)	0 (0)	0 (0)

**Dose-limiting toxicity**	**Description**		**Attribution**	**Dose Level**				
Lymphocyte count decreased grade 3	<500–200/mm3		TMZ	100 mg/kg				

### Pharmacokinetic

The results of the pharmacokinetic assay are presented in Table 3. AGuIX mean AUC increased with dose: 97.5, 174.2, and 201.3 mg.hr/L at 50, 75, and 100 mg/kg, respectively. AGuIX mean Cmax was lower at 50 mg/kg than at 75 and 100 mg/kg. AGuIX mean T_1/2_ ranged from 0.84 (at 75 mg/kg) to 1.41 hr at 100 mg/kg. AGuIX was present in plasma up to 24 h when samples were available. Mean AGuIX CL ranged from 0.43 (at 75 mg/kg) to 0.55 L/h/kg (at 100 mg/kg). AGuIX mean Vss was higher at 100 mg/kg than at 50 and 75 mg/kg: 0.98, 0.77, and 1.64 L/kg at 50, 75, and 100 mg/kg respectively. Mean urinary excretion of AGuIX during the first 6 h after injection ranged from 63 (at 50 mg/kg) to 68 % (at 75 and 100 mg/kg).

**Table 3:** **Pharmacokinetic of Aguix.** Whole blood samples were collected on the second, third and fourth injection with a 9-point kinetic analysis over 24 h. A 6-hour urine collection was also carried out.

AGuIX (mg/kg)	50	75	100
Nb of patients	1	1	6
AUC (mg.hr/L)	97.5 ± 7.8	174.2 ± 3.7	201.3 ± 64.5
Cmax (ng/L)	63.6 ± 5.6	112.3 ± 3.8	83.2 ± 39.4
T_1/2_ (hr)	0.95 ± 0.04	0.84 ± 0.05	1.41 ± 0.3
Urinary excretion (%)	63 ± 4	68 ± 5	68 ± 25
CL (L/h/kg)	0.52 ± 0.05	0.43 ± 0.01	0.55 ± 0.19
Vss (L/kg)	0.98 ± 0.09	0.77 ± 0.05	1.64 ± 0.57

Abbreviations: AUC=area under the plasma concentration/time curve; Cmax = maximum measured plasma concentration; T_1/2_ = half-life; CL=plasma clearance, and Vss = volume of distribution

### Biodistribution

**Fig. 3:**
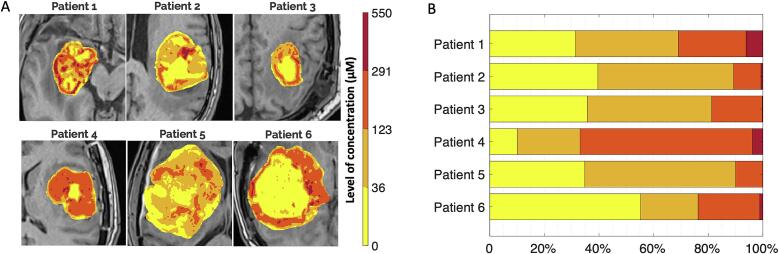
**Level of Gd^3+^ concentration maps (representative of AGuIX concentrations) of patients treated at RP2D (100 mg/kg, n = 6). A.** Representation of the classified voxels of concentration within the glioblastoma region (GBM) according to 4 levels: 0–24, 24–78, 78–198 and 198–550 µM. The selected slices correspond to the slices shown in Fig. 1. The field of view is reduced to 85x85 mm^2^ with a resolution of 0.49 x 0.49 mm^2^. **B.** Breakdown of the quantity of each level, expressed as a proportion of the total GBM volume, for the six patients included in the study.

All patients experienced an increase in signal intensity in GTV on T_1_-weighted MRI after AGuIX injections (Fig. 2A and B). Gd^3+^ concentration maps (representative of AGuIX concentrations) displayed AGuIX accumulation specifically within the GBM (Fig. 2C). The results of the quantification of Gd^3+^ concentrations are summarized in Table 4. The average, minimum, and maximum mean Gd^3+^ concentrations in GTV were 84.9 (±40.3), 54.5, and 160.3 µM. Individual histograms of concentration maps are given in Figures S1 and S2 of Supplementary Materials. Average mean Gd^3+^ concentrations in healthy occipital with matter and vitreous body eyeball (10.9 ± 12.9 and −12.6 ± 7.3 µM, respectively) were within the limits of detection/quantification. Three thresholds were determined for the biodistribution in four levels: 0–36 (low), 36–123 (moderate), 123–291 (high), and 291–550 (very high) µM. The concentration level maps were generated and are shown in Fig. 3. In the concentration level maps, voxels classified at the same level are usually found in the same region of the GTV. Regions with the highest concentration (>123 µM) were predominantly found at the border of the GTV, while regions of lower concentration (<36 µM) were primarily located at the center of the GTV. Analyzing the proportion of each region in relation to the total GTV volume per patient, we noted that regions with high (123–291 µM) or very high (>291 µM) concentration accounted in average for 26.8 ± 19.3 % of the GTV, and up to 66.9 % for patient #4. Regions with low (<36 µM) and moderate (36–123 µM) concentration accounted in average for 34.5 ± 13.3 % and 38.7 ± 12.9 %, respectively. Imaging after the fourth injection of AGuIX exhibited a signal of similar magnitude to the images acquired after the first injection in the region of GBM (Figure S3). The substantial delay between the initial non-enhanced T1 imaging acquired at baseline and the imaging after the fourth injection (19 to 29 days) implied large biological changes induced by treatment, preventing the provision of accurate concentration maps.

**Table 4:** Quantification of Gd^3+^ concentration from AGuIX nanoparticles.

Patient	GBM volume (cc)	Gd^3+^ concentration (µM)					
		GBM				Healthy occipital WM	Vitreous body
		Mean	Quartile 1	Quartile 2	Quartile 3	Mean	Mean
#1	34	100.9 ± 111.3	26.7	68.3	145.6	10.3 ± 10.3	−2.6 ± 9.8
#2	37	54.5 ± 62.4	11.8	50.3	86.5	−12.7 ± 16.8	−19.5 ± 18.1
#3	17	68.3 ± 62.8	16.9	64.1	108.9	8.6 ± 9.5	−6.0 ± 6.2
#4	29	160.3 ± 86.3	94.1	173.6	226.3	24.1 ± 11.2	−10.7 ± 12.6
#5	130	60.0 ± 50.4	27.0	52.7	91.9	13.5 ± 16.5	−19.6 ± 15.5
#6	107	65.6 ± 78.4	18.3	30.9	117.5	21.1 ± 19.7	−17.1 ± 10.4
**Mean**	**59 ± 47**	**84.9 ± 40.3**				**10.9 ± 12.9**	**−12.6 ± 7.3**

Quantification of Gd^3+^ concentration in glioblastoma (GBM), healthy occipital white matter (WM), and vitreous body using the T_1_-weighted MPRAGE MRI acquisition acquired before and few hours post-injection of AGuIX nanoparticles.

## Discussion

The NANO-GBM trial is the first-in-human to evaluate the combination of AGuIX nanoparticles with RT and TMZ in the treatment of newly diagnosed GBM. Here, we present the results of the phase 1b part. In this study, the decision was made to include only patients with a residual macroscopic tumor (biopsy or partial surgery). This decision was made due to the Gadolinium-based formulation of AGuIX nanoparticles, and because we believed it was the best option to demonstrate a radiosensitizing effect. We achieved our primary objective with a RP2D of AGuIX at 100 mg/kg. This result aligns with the finding of the phase 1b clinical trial NANO-RAD [10], which also determined the RP2D of AGuIX to be 100 mg/kg. In the NANO-RAD trial, AGuIX nanoparticles were administered to radiosensitize brain metastases with whole-brain RT alone, with only one AGuIX injection, whereas in the NANO-GBM trial, TMZ-based chemotherapy was also administered, and four AGuIX injections were planned. Here, we report that only 1 out of the 8 patients experienced a DLT (grade 3 lymphopenia), which was not considered related to AGuIX but to TMZ. The only AE considered related to AGuIX was one grade 1 injection site hematoma. This lack of additional toxicity is consistent with the results of the NANO-RAD trial were no DLT related to AGuIX was reported. This is also consistent with the results of the phase 2/3 trial evaluating another type of nanoparticles, namely NBTXR3 nanoparticles (15), in combination with RT in sarcoma. The most common grade 3–4 AE related to NBTXR3 nanoparticles intratumoral injection were injection site pain (4 %), and hypotension (4 %).

Regarding pharmacokinetics, similar to the NANO-RAD study, we found that AGuIX AUC increased with dose level. Plasma elimination T_1/2_ of AGuIX was similar in our study (mean of 1.41 hr at 100 mg/kg) to the NANO-RAD study (mean of 1.21 hr), with a mean urinary excretion of 68 % after 6 h.

Regarding pharmacodistribution, we observed a noticeable increase in signal intensity on MRI after AGuIX injections, indicating the accumulation of AGuIX nanoparticles in tumors. This information is particularly relevant given previous findings that have highlighted a possible correlation between nanoparticle quantity and treatment efficacy [10]. Consequently, this quantification approach holds promise for predicting the impact of AGuIX on individual patients. With this method, we found a selective accumulation of AGuIX nanoparticles exclusively in the tumor tissue, with negligible presence in healthy brain tissue, highlighting the tumor-specificity of AGuIX nanoparticles in the brain, and consequently the absence of possible radiosensitization for surrounding normal tissues. This finding is consistent with the results from the NANO-RAD trial [10]. In the NANO-GBM trial, we also found that the concentration of AGuIX nanoparticles varied significantly between patients and within different regions of the tumor, highlighting the importance of considering the heterogeneity in the vascularization of GBM for treatment planning and outcome prediction. We found that AGuIX accumulation occurred predominantly in the tumor bulk rather than the invasive margin, where the blood–brain barrier remains intact, similar to standard gadolinium contrast imaging. This finding could be of importance as a dose-shrinking correlation had been observed in the previous NANO-RAD trial [10], [13]. Quantitative analysis of the Gd^3+^ concentration unveiled a mean concentration of 84.9 µM across the GBM region, comparable to previous findings in brain metastases [10], [13]. However, the study also revealed concentration peaks reaching up to over 300 µM for several patients, beyond what has already been observed in brain metastases. In future studies, one perspective will be to assess AGuIX levels within tumors through later-stage MRIs. Past findings have demonstrated that following the EPR effect, AGuIX nanoparticles penetrate further into the tumor, remaining detectable up to 7 days later, similar to observations in brain metastases [10]. This implies that the nanoparticles have a tumor half-life of about 2.6 to 3.4 days [17]. Yet, this perspective may oversimplify the situation, as AGuIX nanoparticles distribute throughout various tumor regions and a portion may become immobilized within cells, potentially staying longer than anticipated. For this reason, we believed that 4 AGuIX injections on the first weeks of RT would be sufficient to detect a radiosensitizing effect. Continuous research is deepening our understanding of these dynamics. Given the high levels of AGuIX seen in GBM, we might see a similar or even stronger effect in future observations.

Nanoparticles as AGuIX have a high atomic number (Z). The radiobiological effects of ionizing radiation used in external beam radiotherapy depend on the excitation and ionization of atoms and molecules in the irradiated tissues, and the occurrence of these effects increases with the atomic number (Z) of the target. Consequently, the accumulation of high-Z atoms in the target tissue with nanoparticles, amplifies the energy deposition and radiobiological effects, leading to direct and indirect DNA damage and cell death [6]. In vitro studies on AGuIX have been conducted in collaboration with numerous international teams, including GBM cell lines. In each study, a radiosensitizing effect was demonstrated [18], [19]. Several in vivo studies were also conducted. In all cases, rapid renal elimination and selective tumor accumulation due to the EPR effect were observed. In cases of brain tumors, the nanoparticles accumulate in the tumor after intravenous administration, and MRI contrast enhancement in the tumor remains detectable for at least 24 h post-administration [8]. The radiosensitizing effect of AGuIX has also been proven in vivo, particularly in glioma-bearing animals [6], [7], [8], as well as in models of lung cancer, upper aerodigestive tract cancer, pancreatic cancer, melanoma, and melanoma brain metastases [19]. In an orthotopic rat glioma model, AGuIX doubled the median survival after exclusive irradiation. In the same model, a potential survival benefit was also shown when combining AGuIX with chemoradiotherapy using TMZ [8].

The lack of toxicity related to AGuIX and the extensive dispersion of nanoparticles throughout the GBM are extremely promising. These findings offer robust support for progressing to the randomized phase 2 part of the trial. In this phase, the efficacy of AGuIX radiosensitizing nanoparticles at the RP2D of 100 mg/kg (4 injections) will be assessed in combination with RT and TMZ for the treatment of newly diagnosed GBM.

## CRediT authorship contribution statement

**J. Biau:** Conceptualization, Methodology, Validation, Investigation, Resources, Visualization, Funding acquisition, Writing – original draft. **X. Durando:** Conceptualization, Methodology, Validation, Investigation, Resources, Supervision, Writing – review & editing. **F. Boux:** Methodology, Validation, Formal analysis, Investigation, Resources, Data curation, Writing – original draft. **I. Molnar:** Formal analysis, Data curation, Writing – review & editing. **J. Moreau:** Validation, Investigation, Resources, Funding acquisition, Writing – review & editing. **B. Leyrat:** Investigation, Resources, Writing – review & editing. **F. Guillemin:** Investigation, Resources, Writing – review & editing. **A. Lavielle:** Methodology, Conceptualization, Resources. **Y. Cremillieux:** Methodology, Conceptualization, Resources. **K. Seddik:** Conceptualization, Methodology, Validation, Supervision. **S. Dufort:** Conceptualization, Validation, Data curation, Writing – original draft. **O. De Beaumont:** Conceptualization, Methodology, Supervision, Validation, Writing – review & editing. **E. Thivat:** Conceptualization, Methodology, Validation, Formal analysis, Data curation, Funding acquisition, Writing – review & editing. **G. Le Duc:** Conceptualization, Methodology, Validation, Supervision, Writing – review & editing.

## Declaration of competing interest

The authors declare that they have no known competing financial interests or personal relationships that could have appeared to influence the work reported in this paper.
