# Supplementary material for: NANO-GBM trial of AGuIX nanoparticles with radiotherapy and temozolomide in the treatment of newly diagnosed Glioblastoma: Phase 1b outcomes and MRI-based biodistribution
Source: Clin Transl Radiat Oncol. 2024 Jul 31;48:100833. doi: 10.1016/j.ctro.2024.100833 (PMC11342789; doi:10.1016/j.ctro.2024.100833)
Supplement: Supplementary Data 1 [file mmc1.docx]

**Supplementary Material**

| **Patient** | **Prescribed dose**  (mg.kg^-1^) | **Prepared dose** (mg) | **Relaxivity r_1_ TD-NMR**^(1)^  (s^-1^.mM^-1^_,_ mM of Gd^3+^) | **Delay between end of injection and imaging** |
| --- | --- | --- | --- | --- |
| #1 | 100 | 8000 | 13.1 | 1h44 |
| #2 | 100 | 6700 | 8.5 | 1h19 |
| #3 | 100 | 12900 | 8.5 | 1h00 |
| #4 | 100 | 5700 | 8.5 | 1h15 |
| #5 | 100 | 6100 | 8.5 | 1h32 |
| #6 | 100 | 8400 | 8.5 | 1h05 |

**Table S1: Details of the first injection of AGuIX nanoparticles.**

^(1)^ Time-Domain Nuclear Magnetic Resonance (TD-NMR): Measurements at 37°C with AGuIX concentration of 5 g/L in H_2_O. Various magnetic field strengths, including 20, 40, 60, and 600 MHz, were employed. The relaxivity r1 at 1.5 Tesla (approximately 63.9 MHz) was estimated through regression analysis of these measurements.


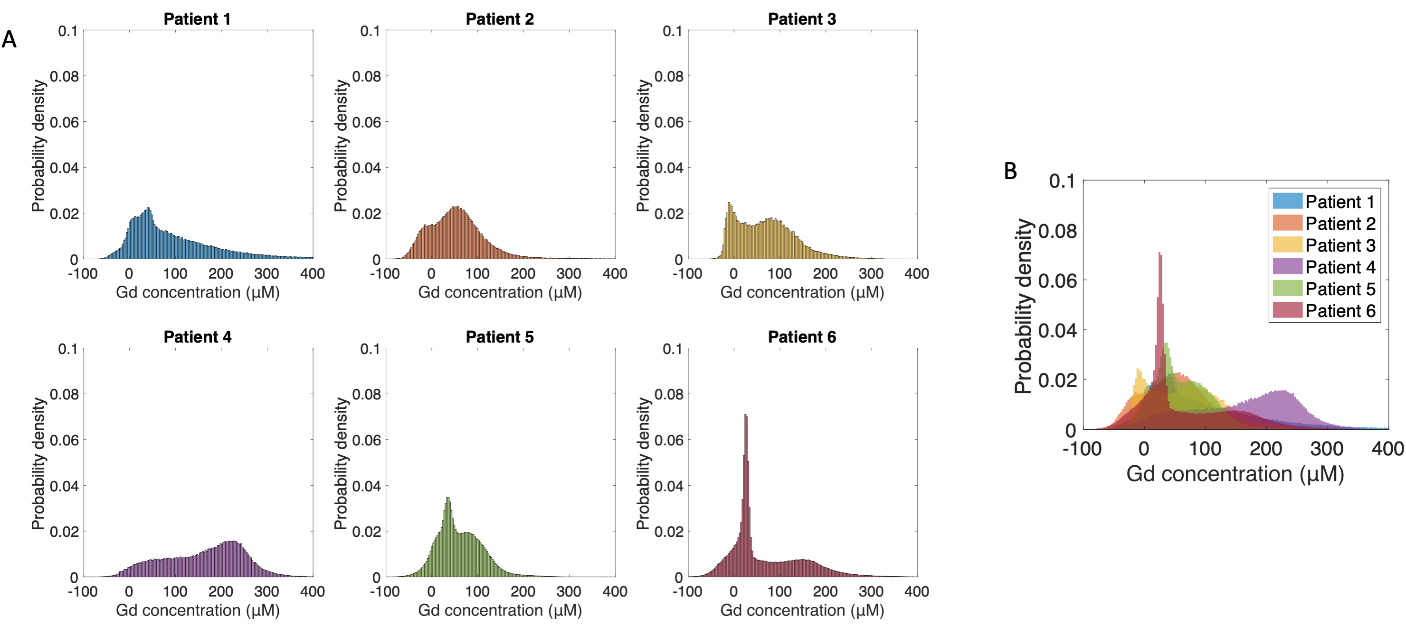


**Figure S1: Histograms of Gd^3+^ concentration in glioblastoma**.

**A.** Individual histograms displaying Gd^3+^ concentration for each patient. **B.** Superimposed histograms for simplified comparison.**
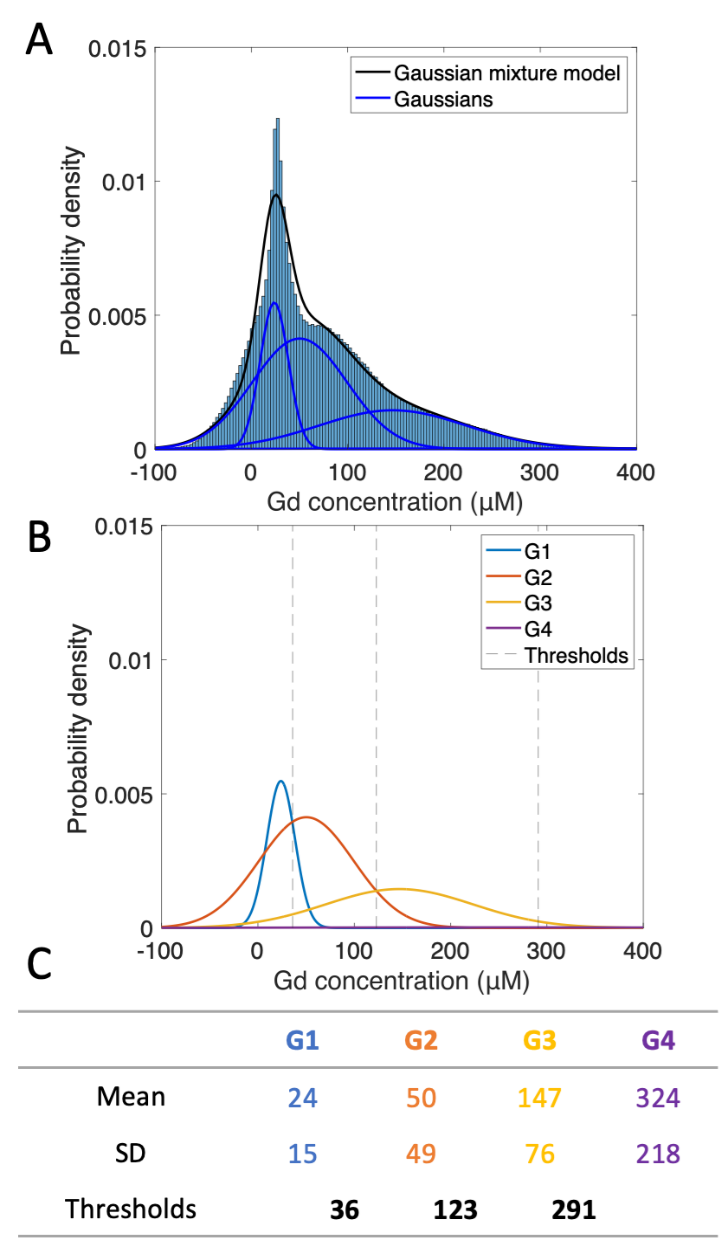
**

**Figure S2: Distribution of Gd^3+^ concentration across glioblastoma and identification of radiosensitizing potential zones.**

**A.** Histogram representing the gadolinium (Gd^3+^) concentration within the glioblastoma region (GBM) for all patients. The histogram is fitted using a 4-Gaussian mixture model, represented by the black curve. **B.** Estimated Gaussians (G1, G2, G3 and G4) of the Gaussian mixture model and the threshold values separating consecutive Gaussians, computed using the Otsu's method. **C.** Summary statistics of the Gaussians, the mean and standard deviation (SD), and threshold values.


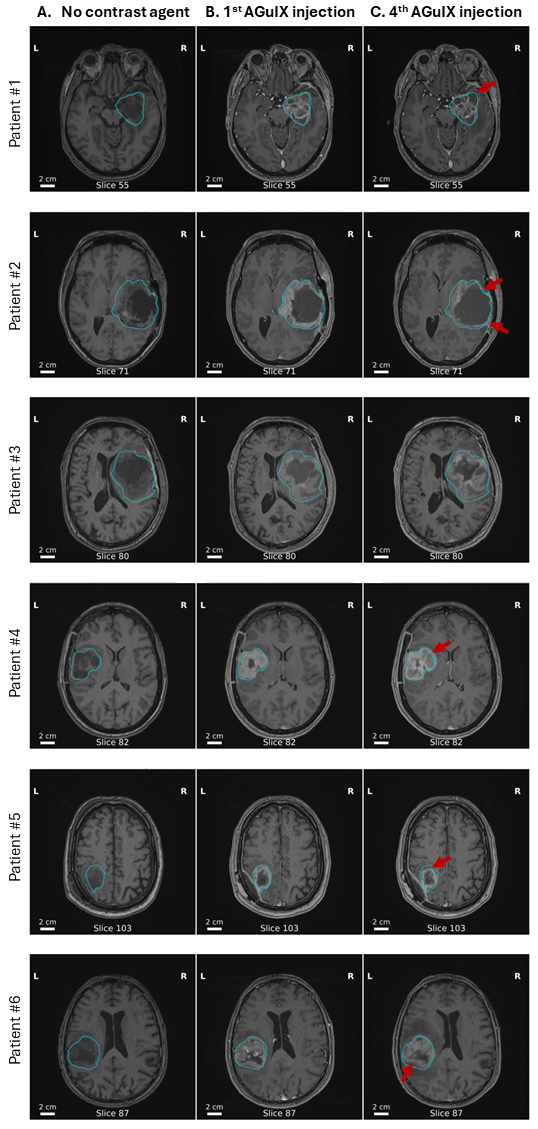


**Figure 3: Registered magnetic resonance imaging at A.** baseline, **B.** after the first AGuIX injection, and **C.** after the fourth AGuIX injection. The slice chosen for each patient is centered on the largest part of the glioblastoma lesion. Blue contours represent the limit of the region of interest used for quantification, and red arrows highlight mismatches between these contours and the lesion on the image acquired after the fourth injection of AGuIX (C).
